# CRISPR/Cas genome editing improves abiotic and biotic stress tolerance of crops

**DOI:** 10.3389/fgeed.2022.987817

**Published:** 2022-09-07

**Authors:** Yangyang Li, Xiuzhe Wu, Yan Zhang, Qiang Zhang

**Affiliations:** ^1^ College of Plant Protection, Shandong Agricultural University, Tai’an, China; ^2^ Hunan Tobacco Research Institute, Changsha, China

**Keywords:** gene editing, CRISRR/cas, crop breeding, abiotic stress, biotic stress

## Abstract

Abiotic stress such as cold, drought, saline-alkali stress and biotic stress including disease and insect pest are the main factors that affect plant growth and limit agricultural productivity. In recent years, with the rapid development of molecular biology, genome editing techniques have been widely used in botany and agronomy due to their characteristics of high efficiency, controllable and directional editing. Genome editing techniques have great application potential in breeding resistant varieties. These techniques have achieved remarkable results in resistance breeding of important cereal crops (such as maize, rice, wheat, etc.), vegetable and fruit crops. Among them, CRISPR/Cas (clustered regularly interspaced short palindromic repeats/CRISPR-associated) provides a guarantee for the stability of crop yield worldwide. In this paper, the development of CRISRR/Cas and its application in different resistance breeding of important crops are reviewed, the advantages and importance of CRISRR/Cas technology in breeding are emphasized, and the possible problems are pointed out.

## Introduction

Genome editing, which involves precise modifications at specific sites in the genome to make desired changes to the DNA sequence. The key breakthrough of genome editing techniques come from the development of site-broken DNA technology. In recent years, with the development of synthetic sequence-specific nuclease (SSN), genome editing techniques have entered a period of rapid development. At present, three types of genome editing tools are widely used by researchers, including zinc finger nuclease (ZFN) (Kim et al., 1996), transcription activator-like effector nuclease (TALEN) (Boch et al., 2009; Christian et al., 2010), and clustered regularly interspaced short palindromic repeats/CRISPR associated (CRISPR/Cas) (Jinek et al., 2012; Cong et al., 2013). ZFN and TALEN have not been widely used due to complicated operation and high failure rate.

The CRISPR/Cas realizes the recognition process through the base complementation between guide RNA and target sequence, which is simple and flexible, and the target site selection only needs to conform to the requirements of protospacer-adjacent motif (PAM) of different systems. Compared to the previous two generations of genome editing techniques, the CRISPR/Cas system is simple, flexible, stable, efficient and easy to transform. These features enabled CRISPR/Cas to quickly replace ZFN and TALEN as the mainstream genome editing techniques. CRISPR/Cas is a defense system, protecting bacteria and archaea from being invaded by mobile genetic elements and bacteriophages (Hille et al., 2018). It is composed of a single-guide RNA (sgRNA), which is a simplified combination of crRNA and tracrRNA, and RNA-guided Cas endonuclease (Hu et al., 2018). During the process of genome editing, Cas endonuclease was recruited by sgRNA to a specific site of the genome to catalyze a DNA double-stranded break (DSB) which can be repaired by diverse DNA repair mechanisms, non-homologous end joining (NHEJ), microhomology-mediated end joining (MMEJ), and homology-directed repairs (HDR), resulting in gene knockout, DNA fragment insertion, deletion, and replacement as specifically required (Hua et al., 2019; Lu et al., 2020). Recently, many efforts have been focused on improving the CRISPR/Cas system to expand the genome-targeting scope of this tools. For example, SpCas9-VRQR, xCas9, and Cas9-NG variants could recognize non-canonical NGA and NG PAM sites in plant (Nishimasu et al., 2018; Ming et al., 2020). SpCas9 orthologues have been identified from *Streptococcus canis* (ScCas9), *Staphylococcus aureus* (SaCas9), *Streptococcus thermophiles* (St1Cas9), and *Brevibacillus laterosporus* (BlatCas9) and have been demonstrated to edit plant genomic loci bearing PAM sequence of NNG, NNGRRT, NNAG AAW, and NNNCND, respectively (Cong and Zhang, 2015; Tan et al., 2020). In addition, the type V Cas12a and Cas12b that isolated from diverse bacterial have been characterized with AT-rich PAM specificity, which were utilized successfully in genome editing of targeted plant (Tang et al., 2017; Wang et al., 2020b).

Since 2013, the CRISPR/Cas has successfully implemented efficient genome editing and regulation in multiple species (Mali et al., 2013; Ran et al., 2015; Xie et al., 2015; Chen et al., 2017). Although CRISPR/Cas has only recently become the preferred tool for genetic manipulation in plants, it has shown great application value in genetic improvement of crops (Wolt et al., 2016). Nowadays, the CRISPR/Cas has been widely used in improving crop yield (Zhou et al., 2019; Cai et al., 2021) and quality (Xing et al., 2020; Xu et al., 2021), enhancing abiotic stress resistance (Nieves-Cordones et al., 2017; Bouzroud et al., 2020) and biotic stress resistance (Ji et al., 2018; Oliva et al., 2019), giving crops herbicide resistance (Zhang et al., 2019b; Liu et al., 2021) and *de novo* crop domestication (Li et al., 2018; Zsögön et al., 2018). Wang et al. (2020a) used CRISPR/Cas9 to edit 25 amino acid sequences conserved at the C-terminal of rice cytokinin-activation enzyme-like gene LONELY GUY (OsLOGL5), and obtained edited lines that significantly increased grain yield under multiple geographical conditions (Wang et al., 2020a). 2-acetyl-1-pyrroline (2AP) is a major source of aroma, and its level can be significantly increased by impaired or deficient function of BETAINE ALDEHYDE DEHYDROGEN-ASE 2 (BADH2), which increases aroma. Using CRISPR/Cas9 to disrupt BADH2 function, the scientists created new rice (*Oryza sativa*), maize (*Zea mays*) and sorghum (*Sorghum bicolor*) germplasm with aroma (Wang et al., 2021a; Tang et al., 2021; Zhang et al., 2022). Alfatih et al. (2020) obtained three independent PARAQUAT TOLERANCE 3 (PQT3) functional deficiency mutants of rice using CRISPR/Cas9, and the germination rate and growth status of the mutants were significantly better than those of the wild type under oxidative stress and salt stress (Alfatih et al., 2020). 5-enolpyruvylshikimate-3-phosphate synthase (EPSPS) is a key enzyme involves in the synthesis pathway of aromatic amino acids, and is the target of glyphosate, a broad-spectrum and highly effective pesticide. Wang et al. (2021a) used a CRISPR/Cas9-mediated ho-mology directed repair (HDR) strategy to successfully replace endogenous EPSPS with EPSPSmTIPS and EPSPSmLFGAAGMCRL in rapeseed, and obtained a new line with stable inheritance and glyphosate-resistant rapeseed (Wang et al., 2021b). Yu et al. (2021) accelerated *de novo* domestication of wild rice by using CRISPR/Cas9 multi-gene editing targeting genes for important agronomic traits (Yu et al., 2021).

In recent years, with the development of industry and frequent occurrence of extreme climate, the natural environment has gradually developed towards the unsuitable direction for the growth of crops. Under the influence of abiotic stresses such as low temperature (Shi et al., 2018), high temperature (Tang et al., 2020), drought (Gupta et al., 2020), saline-alkali (Ismail and Horie, 2017), heavy metal (Chauhan et al., 2020), and biotic stresses including fungal, bacterial, viral diseases (Li et al., 2019) and insect pests (Liu et al., 2016), the yield and quality of crops are reduced. Traditional crossbreeding, mutagenesis breeding and other breeding methods can not meet the requirements of resistance breeding (Zhang et al., 2017). CRISPR/Cas can be used for directional improvement of crops and greatly shorten the breeding life, which has become the mainstream technology of resistance breeding at present. This paper reviews the application of CRISPR/Cas in crop re-sistance gene improvement, and puts forward the possible problems and challenges.

## CRISPR/Cas gene editing improves abiotic stress tolerance of crops

Abiotic stresses such as salinity, drought, extreme temperature and heavy metals are important factors affecting plant growth and development, which can lead to 50% crop yield reduction (Liu et al., 2022). It is essential to generate crop types with greater adaptability for growth under a variety of environmental conditions in such circumstances. Though traditional breeding increases production to a large extent, it has the drawback of losing genetic variety and fitness (Wolter et al., 2019). In addition to being time-consuming, its reliance on natural allelic variants makes it difficult to create the desired characteristic and ensure the sustainability of production (Gao, 2018). Herein, we reviewed CRISPR/Cas-mediated crop plant editing to address the remarkable problem of various abiotic stressors in this paper (Table 1).

**TABLE 1:** Genes targeted by CRISPR/Cas system for imparting tolerance against abiotic stress.

Stress	Crop	The name of target gene	References
Salinity	Tomato (*Solanum lycopersicum*)	*HYBRID PROLINE-RICH PROTEIN 1* (*SlHyPRP1*)	Tran et al. (2021)
	Tomato (*Solanum lycopersicum*)	*Auxin Response Factor 4* (*SlARF4*)	Bouzroud et al. (2020)
	Rice (*Oryza sativa*)	*BASIC HELIX-LOOP-HELIX 024 (OsbHLH024)*	Alam et al. (2022)
	Rice (*Oryza sativa*)	*RESPONSE REGULATORS 22* (*OsRR22*)	Zhang et al. (2019a), Han et al. (2022)
	Rice (*Oryza sativa*)	*RELATED TO ABI3/VP1 2* (*OsRAV2*)	Liu et al. (2020b)
	Rice (*Oryza sativa*)	*DROUGHT AND SALT TOLERANCE* (*OsDST*)	Santosh Kumar et al. (2020)
	Rice (*Oryza sativa*)	*NAM, ATAF and CUC 041* (*OsNAC041*)	Wang et al. (2016b)
	Rice (*Oryza sativa*)	*OsmiR535*	Yue et al. (2020)
	Barley (*Hordeum vulgare*)	*INOSITOL TRISPHOSPHATE 5/6 KINASES 1* (*HvITPK1*)	Vlčko and Ohnoutková, (2020)
Drought	Maize (*Zea mays*)	*AUXIN-REGULATED GENE INVOLVED IN ORGAN SIZE 8* (*ZmARGOS8*)	Shi et al. (2017)
	Wheat (*Triticum aestivum*)	*DEHYDRATION RESPONSIVE ELEMENT BINDING PROTEIN 2* (*TaDREB2*)	Kim et al. (2018)
	Wheat (*Triticum aestivum*)	*ETHYLENE-RESPONSE FACTOR 3* (*TaERF3*)	Kim et al. (2018)
	Rice (*Oryza sativa*)	*ENHANCED RESPONSE TO ABA1* (*OsERA1*)	Ogata et al. (2020)
	Rice (*Oryza sativa*)	*OsDST*	Santosh Kumar et al. (2020)
	Rice (*Oryza sativa*)	*PYRABACTIN RESISTANCE-LIKE 9* (*OsPYL9*)	Usman et al. (2020)
	Rice (*Oryza sativa*)	*SEMI-ROLLED LEAF 1* (*SRL1*) and *SEMI-ROLLED LEAF 2* (*SRL2*)	Zeng et al. (2019)
	Tomato (*Solanum lycopersicum*)	*GA-INSENSITIVE DWARF1 1* (*SlGID1*)	Illouz-Eliaz et al. (2020)
	Tomato (*Solanum lycopersicum*)	*LATERAL ORGAN BOUNDARIES DOMAIN 40* (*SlLBD40*)	Liu et al. (2020a)
Low temperature	Rice (*Oryza sativa*)	*PIN-FORMED 5b* (*OsPIN5b*)	Liao et al. (2019)
	Rice (*Oryza sativa*)	*GRAIN SIZE* (*GS3*)	Liao et al. (2019)
	Rice (*Oryza sativa*)	*V-MYB AVIAN MYELOBLASTOSIS VIRAL ONCOGENE HOMOLOG 30* (OsMYB30)	Liao et al. (2019)
High temperature	Tomato (*Solanum lycopersicum*)	*MITOGEN-ACTIVATED PROTEIN KINASES 3* (*SlMAPK3*)	Yu et al. (2019)
	Rice (*Oryza sativa*)	*PYRABACTIN RESISTANCE-LIKE 1/4/6* (*OsPYL1/4/6*)	Miao et al. (2018)
Cadmium	Rice (*Oryza sativa*)	*NATURAL RESISTANCE-ASSOCIATED MACROPHAGE PROTEIN 5* (*OsNRAMP5*)	Chang et al. (2020), Chu et al. (2022)
	Rice (*Oryza sativa*)	*LOW-AFFINITY CATION TRANSPORTER 1* (*OsLCT1*)	Chang et al. (2020)
	Rice (*Oryza sativa*)	*NATURAL RESISTANCE-ASSOCIATED MACROPHAGE PROTEIN 1* (*OsNRAMP1*)	Chang et al. (2020), Chu et al. (2022)
Arsenic	Rice (*Oryza sativa*)	*ARSENITE-RESPONSIVE MYB1* (*OsARM1*)	Wang et al. (2017)
Caesium	Rice (*Oryza sativa*)	*HIGH-AFFINITY POTASSIUM TRANSPORTER 1 (OSHAK1)*	Nieves-Cordones et al. (2017)

It has been reported that 7% of the Earth’s land and 20% of arable land were affected by salinization, and the situation was only going to deteriorate (Al Murad et al., 2020). Salt stress induces osmotic stress, ion stress and secondary stress in plants (Yang and Guo, 2018), which reduces yield and quality of crops (Siddiqui et al., 2017). In tomato plants, the exact deletion of *SlHyPRP1* negative-response domain(s) significantly enhanced the salinity tolerance at both of the germination and vegetative stages (Tran et al., 2021). Alam et al. (2022) used CRISPR/Cas9 system to knock out *OsbHLH024* gene in rice and enhance the expression of ion transporter gene *OsHKT1;3*, *OsHAK7*, and *OsSOS1*, enhancing salt tolerance of rice (Alam et al., 2022). Mutation of *OsRR22* gene induced by CRISPR/Cas9 enhanced salt tolerance of rice without changing other agronomic traits (Zhang et al., 2019a; Han et al., 2022). *OsRAV2* was successfully mutated using CRISPR/Cas9, and the mutant had higher survival viability under salt stress (Liu et al., 2020b). In addition, using CRISPR/Cas9 technology to knock out rice *OsDST* (Santosh Kumar et al., 2020), *OsNAC041* (Wang et al., 2016b) and *OsmiR535* (Yue et al., 2020)], barley *HvITPK1* (Vlčko and Ohnoutková, 2020) and tomato *SlARF4* (Bouzroud et al., 2020) can also effectively improve the ability of crops to resist salt stress.

Drought stress is the main cause of serious loss of yield and productivity of major crops and poses the greatest threat to global food security (Joshi et al., 2020). Using CRISPR/Cas system, the natural *ARGOS8* promoter sequence of maize was replaced by *GOS2* promoter to improve the yield of maize under drought stress in field (Shi et al., 2017). CRISPR/Cas9-mediated mutagenesis of *OsERA1* resulted in great drought stress tolerance in rice (Ogata et al., 2020). Drought resistance of wheat was improved by CRISPR/Cas editing of wheat *TaDREB2* and *TaERF3* (Kim et al., 2018). Santosh Kumar et al. (2020) used CRISPR/Cas9 to edit *OsDST* gene to obtain the mutant of indica mega rice cultivar MTU1010, with wider leaves, lower stomatal density and enhanced leaf water retention ability under drought stress (Santosh Kumar et al., 2020). Usman et al. (2020) found that the *ospyl9* mutant created by CRISPR/Cas9 could improve drought tolerance and yield of rice (Usman et al., 2020). CRISPR/Cas9 induced *SRL1* and *SRL2* gene mutations in rice to achieve the curled leaves phenotype and drought tolerance by changing expression patterns of protein and scavenging of reactive oxygen species (Liao et al., 2019). Tomato plants with high leaf water content were obtained under drought conditions using CRISPR/Cas9 to modify GID1, and tomato drought resistance was effectively increased (Illouz-Eliaz et al., 2020). In addition, CRISPR/Cas9-mediated *SlLBD40* gene mutation also significantly enhanced drought resistance of tomato (Liu et al., 2020a).

Cold stress, which includes chilling (<20°C) and freezing (<0°C) temperatures, inhibited growth and development of plants, and seriously restricts plant spatial distribution and agricultural productivity (Ding et al., 2020). Low temperature directly inhibits plant metabolic response and induces osmotic stress, oxidative stress and other stress. Zeng et al. showed that the *ospin5b* mutant, *gs3* mutant and *osmyb30* mutant created by CRISPR/Cas9 increased spike length, grain size and cold tolerance (Zeng et al., 2019). High temperature affects the whole growth cycle of crops, especially in the heat sensitive period such as early establishment, flowering and gametophytogenesis (Jagadish et al., 2021). Compared with the wild type, the CRISPR/Cas9-mediated *slmapk3* mutant maintained reactive oxygen species homeostasis by regulating the expression of antioxidant enzymes and *HSPs*/*HSFs* genes, enhancing the high temperature tolerance of tomato plants (Yu et al., 2019). CRISPR/Cas9 editing was used to make *pyl1/4/6* triple knockout rice. The mutant showed a greater yield, higher temperature tolerance, and less germination before harvest than the wild variety (Miao et al., 2018). Heavy metal toxicity is one of the most destructive abiotic stress.

Heavy metals cause serious damage to plant growth and yield and are the main problem of sustainable agricultural development. It has adverse effects on plant physiology and biochemistry through osmotic stress, ion imbalance, oxidative stress, membrane tissue disorder, cytotoxicity and metabolic homeostasis (Hoque et al., 2021). Heavy metals accumulated in plants will cause serious harm to human health after ingestion (Kaur et al., 2021). Knockdown of *OsNramp5* and *OsLCT1* by CRISPR/Cas9 reduces cadmium (Cd) accumulation in rice (Songmei et al., 2019). *OsNRAMP1* was knocked out using CRISPR/Cas9, which resulted in lower Cd and plumbum (Pb) levels in rice grains (Chang et al., 2020; Chu et al., 2022). At the same time, the function of *OsNRAMP5* and *OsNRAMP1* to reduce Cd accumulation is not redundant. Wang et al. (2017) inhibited the absorption and transport of arsenic in rice by eliminating an R2R3 MYB transcription factor *OsARM1* by CRISPR/Cas9 (Wang et al., 2017). To create low Caesium (Cs) rice plants, Nieves-Cordones et al. (2017) used the CRISPR/Cas to inactivate the K^+^ transporter *OsHAK1* (Nieves-Cordones et al., 2017).

## CRISPR/Cas gene editing improves biotic stress tolerance of crops

Biotic stresses, such as viral, fungal, and bacterial infections, account for 20–40% of global agricultural output losses (Walker, 1984). In order to address the food crisis, conferring pathogen resistance to host plants can lessen the impact of disease on crop productivity (Borrelli et al., 2018). So far, scientists have obtained plants that are highly resistant to fungal, bacterial and viral diseases, as well as insects, through CRISPR/Cas9 knockout (Chen et al., 2019) (Table 2).

**TABLE 2:** Genes targeted by CRISPR/Cas for imparting tolerance against biotic stress.

Stress		Crop	The name of target gene	References
Fungus disease	Powdery mildew	Tomato (*Solanum lycopersicum*)	*MILDEW RESISTANT LOCUS O* (*SlMLO*)	Nekrasov et al. (2017)
		Wheat (*Triticum aestivum*)	*TaMLO-A1*, *TaMLO-B1* and *TaMLO-D1*	Wang et al. (2014)
		Grapevine (*Vitis vinifera*)	*VvMOL3*	Wan et al. (2020)
		Tomato (*Solanum lycopersicum*)	*POWDERY MILDEW RESISTANCE 4* (*SlPMR4*)	Santillán Martínez et al. (2020)
	Rice Blast	Rice (*Oryza sativa*)	*OsERF922*	Wang et al. (2016a)
		Rice (*Oryza sativa*)	*SUBUNIT OF THE EXOCYST COMPLEX 3A* (*OsSEC3A*)	Ma et al. (2018)
		Rice (*Oryza sativa*)	*Pi21* and *Bsr-d1*	Nawaz et al. (2020), Zhou et al. (2022)
	Late blight	Tomato (*Solanum lycopersicum*)	miR482b and miR482c	Hong et al. (2021)
	Gray mould	Tomato (*Solanum lycopersicum*)	*PECTATE LYASE (SlPL)*	Silva et al. (2021)
Bacterial disease	Bacterial blight	Rice (*Oryza sativa*)	*SUGARS WILL EVENTUALLY BE EXPORTED TRANSPORTER 13* (*OsSWEET13*)	Zhou et al. (2015)
	Citrus bacterial canker	Orange (*Citrus sinensis*)	*LATERAL ORGAN BOUNDARY 1* (*CsLOB1*)	Jia et al. (2016), Peng et al. (2017)
	Bacterial leaf spot disease	Tomato (*Solanum lycopersicum*)	*JASMONATE ZIM-DOMAIN 2* (*SlJAZ2*)	Ortigosa et al. (2019)
Virus disease	Cucumber vein yellowing virus	Cucumber (*Cucumis sativus*)	*EUKARYOTIC TRANSLATION INITIATION FACTOR 4E* (*eIF4E*)	Chandrasekaran et al. (2016)
	Zucchini yellow mosaic virus	Cucumber (*Cucumis sativus*)	*eIF4E*	Chandrasekaran et al. (2016)
	Papaya ring spot mosaic virus-W	Cucumber (*Cucumis sativus*)	*eIF4E*	Chandrasekaran et al. (2016)
	Rice tungro spherical virus	Rice (*Oryza sativa*)	*eIF4G*	Macovei et al. (2018)
	Tomato mosaic virus	Tomato (*Solanum lycopersicum*)	*DICER-LIKE 2b (SlDCL2b)*	Wang et al. (2018a)
	Potato virus X	Tomato (*Solanum lycopersicum*)	*SlDCL2a* and *SlDCL2b*	Wang et al. (2018b)
Insect disease	Plant hopper	Rice (*Oryza sativa*)	*CYTOCHROME P450 71A1* (*OsCYP71A1*)	Lu et al. (2018)
	Stem borer	Rice (*Oryza sativa*)	*OsCYP71A1*	Lu et al. (2018)
	Common cutworm	Soybean (*Glycine max*)	*CALCIUM-DEPENDENT PROTEIN KINASE 38* (*GmCDPK38*)	Li et al. (2022)

Fungus disease is a kind of devastating disease in crops, among which powdery mildew seriously affects crop productivity. CRISPR/Cas9 was used to knock out all three *TaMLO* alleles in wheat, and wheat plants with enhanced powdery mildew resistance were obtained (Wang et al., 2014). Similarly, CRISPR/Cas9-mediated knockdown of *SlMLO* and *VvMOL3* made tomato (Nekrasov et al., 2017) and grape (Wan et al., 2020) resistant to powdery mildew. In addition, CRISPR/Cas9-mediated *SlPMR4* mutation also significantly increased tomato powdery mildew resistance, but could not completely immune (Santillán Martínez et al., 2020). Rice blast is a destructive fungal disease. CRISPR/Cas9 was used to enhance resistance to rice blast disease by interrupting the *OsERF922* and *OsSEC3A* genes in rice (Wang et al., 2016a; Ma et al., 2018). Among them, other agronomic traits of *oserf922* mutant did not change (Wang et al., 2016a), while SA content in *ossec3a* increased, resulting in dwarfing (Ma et al., 2018). CRISPR/Cas9-induced rice *Bsr-d1* and *Pi21* mutations could also cause partial resistance to rice blast, but the effect was not as strong as *oserf922* (Nawaz et al., 2020; Zhou et al., 2022). Tomato late blight is a serious tomato fungal disease caused by *Phytophthora infestans*, which mainly affects tomato yield. miRNAs can enhance plant resistance by inhibiting their target genes. *miR482b* and *miR482c* were simultaneously knocked out by multiple editing systems, and double mutants were found to have higher resistance than single mutants, revealing a new mechanism by which miRNAs regulate fungal resistance (Hong et al., 2021). Furthermore, Silva et al. (2021) discovered that when the *PL* was knocked out by CRISPR/Cas9, the incidence of gray mold infection in tomato fruits was significantly reduced (Silva et al., 2021).

Of all the bacterial species on earth, hundreds can cause disease in plants, often exposing multiple disease symptoms (Schloss and Handelsman, 2004). Plant pathogenic bacteria are difficult to control due to the difficulty of detecting disease before it appears and the lack of effective pesticides. CRISPR/Cas9 modification of plant genomes has been found to improve crop resistance to bacterial diseases. For example, *OsSWEET13* is a susceptibility (S) gene that codes for a sucrose transporter that plays an important role in the interaction between plant and pathogen. PthXo2, an effector protein produced by *X. oryzae*, causes *OsSWEET13* expression in the host and, as a result, susceptibility. In rice plants, knocking down the promoter *OsSWEET13* resulted in bacterial blight resistance (Zhou et al., 2015). Citrus bacterial canker (CBC) is the most widespread bacterial disease in citrus, which was caused by *Xanthomonas citri* subspecies *citri*. Jia et al. (2016) generated CBC-resistant mutants by editing the promoter sequence of the *CsLOB1* gene in Duncan grapefruit (Jia et al., 2016). Meanwhile, Peng et al. (2017) also reported that CRISPR/Cas9 targeted modification of citrus susceptible gene *CsLOB1* promoter EBE_PthA4_ combined with the original to improve the resistance of Wanjincheng orange (*Citrus sinensis* Osbeck) to citrus canker disease (Peng et al., 2017). *Pseudomonas Syringae* is the cause of bacterial leaf spot disease. It induces stomatal opening of plants by releasing coronatine, which is conducive to bacterial infection. The Jasmonate-ZIM domain protein is a COR coreceptor, and *SlJAZ2* is edited by CRISPR/Cas9 to lack JAZ domain, resulting in resistance to bacterial leaf spot disease (Ortigosa et al., 2019). In addition, the CRISPR/Cas9 technology provides a strategy for the creation of multiple resistant materials that induce mutations in the acetylegenase-encoding genes *ACER1a* and *ACET1b* that show increased resistance to fungal and bacterial pathogens (Jeon et al., 2020). CRISPR/Cas9-induced mutations in tomato susceptibility gene *SlDMR6-1* confer resistance to different types of pathogens, including bacteria, oomycetes and fungi (Thomazella et al., 2021). CRISPR/Cas9-mediated *osnramp1* mutants increased hydrogen peroxide (H_2_O_2_) content and superoxide dismutase (SOD) activity, but decreased catalase (CAT) activity, showing broad-spectrum resistance to bacteria and fungi (Chu et al., 2022).

A number of economically important staples and specialty crops are threatened by plant viruses. According to the nature of their genomes, they are divided into six major groups: single-stranded DNA (ssDNA), double-stranded DNA (dsDNA)viruses with no plant viruses in this group, double-stranded RNA (dsRNA), reverse-transcribing viruses, positive sense single-stranded RNA (ssRNA+) viruses and negative sense single-stranded RNA (ssRNA) (Roossinck et al., 2015). Studies using CRISPR-edited plants for virus resistance have focused on ssDNA geminivirus genomes (Ali et al., 2015; Baltes et al., 2015; Ji et al., 2015). It includes many kinds of plant viruses that causes worldwide crop losses, affecting many important families, including Euphorbiaceae, Cucurbitaceae, Malvaceae, Solanaceae, and Fabaceae (Zaidi et al., 2016). By rolling-circle amplification or recombination-mediated replication, the virus genome replicates itself *via* a dsDNA replicative form (Hanley-Bowdoin et al., 2013). In economic terms, *Begomovirus* is the most important genus of geminiviruses. *Begomovirus* infect dicotyledonous plants primarily through the sweet tobacco/potato/silverleaf whitefly (*Bemisia tabaci*) and are found attached to phloem of plants (Gilbertson et al., 2015). The genome is composed of one (A, monopartite) or two (A and B, bipartite) components, which contain a common 220-bp region (Fondong, 2013). At first, Baltes et al. (2015) and Ji et al. (2015) reported on resistance to geminiviruses, beet severe curly top virus (BSCTV) and bean yellow dwarf virus (BeYDV) in model plants *Nicotiana benthamiana* and *Arabidopsis* Baltes et al., 2015; Ji et al., 2015). Ji et al. (2015) identified 43 possible sgRNA/Cas9 targets within the coding and non-coding domains of the BSCTV genome (Ji et al., 2015). Each sgRNA/Cas9 construct reduced virus content to varying degrees in inoculated leaves. And, the plants with the highest expressing levels of Cas9 and sgRNAs seem to be more resistance to virus infection. Similarly, Baltes et al. (2015) utilized 11 sgRNAs targeting Rep motifs, Rep-binding sites, hairpins, and the nonanucleotide sequence of BeYDV to achieve similar results (Baltes et al., 2015). A CRISPR/Cas9 approach was also used for enhancing resistance to begomovirus in two recent studies (Ali et al., 2015; Ali et al., 2016). The CRISPR/Cas9 systems were expressed in the host cell nucleus, and the viral genome was targeted and cleaved during replication in both studies. The sgRNA molecules developed by Ali et al. (2015) were delivered into *N. benthamiana* plants overexpressing the Cas9 endonuclease *via* a tobacco rattle virus (TRV) vector (Ali et al., 2015). SgRNAs target different coding and non-coding sequences of the tomato yellow leaf curl virus (TYLCV), including the RCRII motif of the replication protein (Rep), the capsid protein (CP) and the intergenic region (IR). The sgRNAs that target stem-loop invariant sequences in the IR caused a significant reduction in viral replication and accumulation but did not interfere with TYLCV genome sequences. Bipartite Merremia mosaic virus (MeMV) and the monopartite beet curly top virus (BCTV) have same stem-loop sequence in the IR. Therefore, CRISPR/Cas9 system was designed to target this sequence simultaneously. The results demonstrated that a sgRNA specific for conserved sequences from multiple viral strains can be used to realize mixed infection immunity. Furthermore, Different CRISPR/Cas9 tools were designed to target different coding and non-coding sequences of MeMV, cotton leaf curl Kokhran virus (CLCuKoV), and different severe and mild strains of TYLCV (Ali et al., 2016). Researchers found that when the viral coding regions were edited by sgRNA/Cas9 complex, virus variants that were able to replicate and escape CRISPR/Cas9 were generated. In contrast, no new variants were found in plants carrying sgRNAs targeting the IR sequences in *N. benthamiana* plants. A second technique for achieving viral disease resistance entails altering plant genes that provide virus resistance qualities, segregating the CRISPR/Cas9 tool, and releasing non-transgenic mutants into the field (Chandrasekaran et al., 2016; Pyott et al., 2016; Macovei et al., 2018). Plant host factors, such as the eukaryotic translation initiation factors *eIF4E*, *eIF(iso)4E*, and *eIF4G*, are required by RNA viruses to maintain their life cycle (Sanfaçon, 2015). By modifying two different sites of the host susceptibility gene *eIF4E* with CRISPR/Cas9, Chandrasekaran et al. (2016) were able to develop cucumber plants that were resistant to potyviruses. Homozygous *eif4e* mutants demonstrated protection to viruses from the Potyviridae family, such as zucchini yellow mosaic virus (ZYMV), cucumber vein yellowing virus (CVYV), and papaya ring spot mosaic virus-W (PRSV-W). However, heterozygous knockout plants and nonmutant plants showed no resistance to these viruses. Macovei et al. (2018) used mutagenesis of *eIF4G* alleles in rice plants to establish novel sources of resistance to rice tungro spherical virus (RTSV) (Macovei et al., 2018). Furthermore, after inoculation with RTSV, agronomic parameters, e.g., plant height and grain production of the edited rice plants were improved compared to that the wild-type. In tomato plant, CRISPR/Cas9 was used to edit *SlDCL2b* with the highest expression in four *DCL2* subfamilies (*SLDCL2a*-*SLDCL2d*), which significantly enhanced tomato resistance to tomato mosaic virus (ToMV) (Wang et al., 2018a). Wang et al. (2018a) discovered that editing *SlDCL2a* and *SlDCL2b* at the same time increased tomato resistance to potato virus X (PVX) and tobacco mosaic virus (TMV) (Wang et al., 2018b).

Pests is thought to be responsible for 20–40% of global crop output loss (Douglas, 2018). Pests adversely affect crop yield and quality through direct damage and transmission of plant diseases (Oerke, 2006). In recent years, agricultural losses caused by pests have increased as the climate has warmed (Deutsch et al., 2018), but widespread use of pesticides can have a negative impact on the environment. Therefore, there is an urgent need for a safe and effective method to control the occurrence of insect pests on crops. The level of salicylic acid levels was raised when the serotonin biosynthesis was prevented by disrupting *OsCYP71A1*, which results in greater resistance to plant hoppers and stem borers in rice (Lu et al., 2018). Li et al. (2022) showed that the *gmcdpk38* mutant with Hap3 knockout using CRISPR/Cas9 showed high resistance to common cutworm (Li et al., 2022).

## Conclusion and future prospects

**FIGURE 1:**
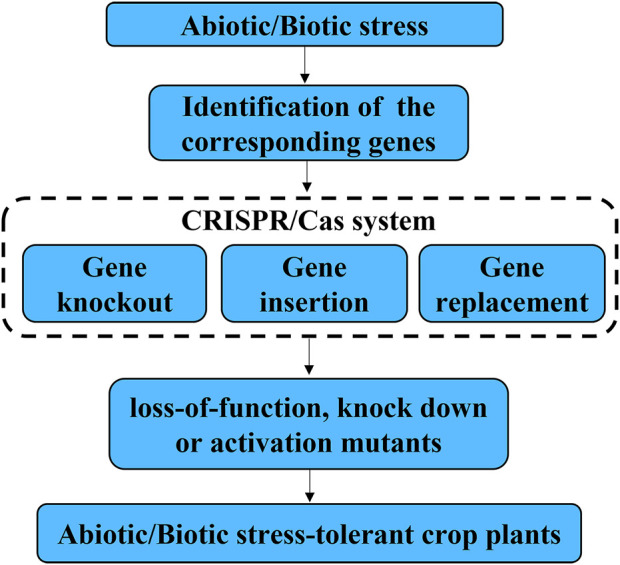
Review of the applications of CRISPR/Cas systems in improving biotic and abiotic stress tolerance of crop. CRISPR-Cas9 system can be used for gene knockout, gene insertion and gene replacement, resulting in loss-of-function, knock down or activation mutants, which can lead to generation of abiotic/biotic stress-tolerant crop plants.

Traditional plant breeding, such as conventional intergeneric crosses and chemical/physical mutagenesis are non-specific. Moreover, introgression of beneficial traits into an elite variety is often accompanied by introgression of non-target traits because of linkage drag. Therefore, it is a long-term effort for the development of new cultivars using traditional breeding methods, especially it is time-consuming of backcross to segregate the unwanted changes in their offspring (Hartung and Schiemann, 2014). In contrast, CRISPR/Cas9 can accelerate plant breeding by modify genomes rapidly in a precise and predictable manner. Because of its efficiency, simplicity, and versatility, CRISPR/Cas9 has recently become a popular tool for genome editing and has been widely used in crop resistance breeding (Wolter et al., 2019). CRISPR-Cas9 system can be used for gene knockout, gene insertion and gene replacement, resulting in loss-of-function, knock down or activation mutants, which can lead to generation of abiotic/biotic stress-tolerant crop plants (Figure 1). Meanwhile, the availability of the genome sequence of crops allows scientists to precisely design its genome, which facilitates the application of CRISPR/Cas9 in resistance breeding (Jackson et al., 2011). Firstly, the major genes controlling important traits of crops have not been identified, which limits the application of CRISPR/Cas in plant genetic engineering breeding (Jin et al., 2019). Secondly, pathogens keep to modify its genome though evolution to break the already available resistance obtained by CRISPR/Cas gene editing. Therefore, it is required to design new variants every few years and insert them into the plants(Ahmad et al., 2020). Thirdly, many genes are represented by multi-gene families with functional redundancy, making it difficult to produce resistance phenotype by knocking-out a single gene (Li et al., 2013). It is required to develop powerful CRISPR-Cas tools to realize multiplex genome editing.

Off-target effect is another major limitation of CRISPR-Cas system. Although much work has been done to optimize CRISPR/Cas system and improve its specificity, there is still no way to completely avoid editing individuals off-target (Pattanayak et al., 2013; Fu et al., 2014; Kleinstiver et al., 2016; Tang et al., 2019). Therefore, the potential off-target risk cannot be ignored when using CRISPR/Cas for genome editing. For gene function studies, in order to exclude misjudgment of results caused by off-target phenomenon, association analysis between genotype and phenotype should be carried out in multiple independently edited individuals to determine whether phenotypic changes are caused by mutations in target genes. In addition, one big challenge in crop breeding is efficient delivery of CRISPR/Cas components into reproductive cells. In the case of plants that can be transformed, foreign genes can be introduced into their reproductive cells by genetic transformation methods in a quite effective way. However, the associated tissue culture and regeneration steps are time consuming and complex. Furthermore, many crops are recalcitrant or extremely difficult to transform. For gene editing to be applied to all plants, we need technology that can deliver gene editing reagents independent of tissue culture and plant regeneration.

Despite the overwhelming benefits of CRISPR/Cas system for crop improvement, regulatory policies that classify gene-edited goods as GMOs may prevent their use in some nations (Callaway, 2018). However, from a scientific point of view, the mutants obtained by CRISPR/Cas are exactly the same as those obtained by natural mutation or conventional mutagenesis after removing the transgenic label. We think more publicity should be given in this regard to dispel the prejudice of most people. On January 24, 2022, the Ministry of Agriculture and Rural Affairs of The People’s Republic of China issued the 《Guidelines for Safety Evaluation of Gene-edited Plants for Agricultural Use (Trial)》 (http://www.moa.gov.cn/ztzl/zjyqwgz/sbzn/202201/t20220124_6387561.htm). This guideline mainly applies for safety evaluation of gene-edited plants without introducing exogenous genes according to different risk levels to apply for production and application safety certificates. The release of this guideline provides a basis for the standardized development of gene-edited crops and a reference for further deregulation of gene-edited crops in the future.

CRISPR and other gene editing technologies have already made significant gains in crop breeding, and we expect that this is just the beginning, with many more exciting developments to follow. With the development of second-generation sequencing, gene editing technology and target analysis technology based on high-throughput sequencing method have a solid technical foundation, and the acquisition of high-throughput big data has become more common, convenient and affordable, which will greatly promote the application of CRISPR/Cas in crop genetic improvement.
